# Anti-peptide antibodies in the diagnosis of malaria

**DOI:** 10.1186/1475-2875-9-S2-P12

**Published:** 2010-10-20

**Authors:** J P Dean Goldring, Ramona Hurdayal, David Choveaux

**Affiliations:** 1 Ikechukwu Achilonu Biochemistry, University of KwaZulu-Natal, Pietermaritzburg, 3209, South Africa

## 

Lactate dehydrogenase is one of the antigens targeted in lateral flow immunochromatographic rapid diagnostic tests for the diagnosis of malaria. Unique diagnostic peptide targets based on the respective amino-acid sequences to differentiate between *P. falciparum* and *P. vivax* lactate dehydrogenase were chosen, synthesized and coupled to rabbit albumin carrier. Anti-peptide antibodies were raised in chickens against the peptides and against each of the recombinant proteins and affinity purified. An antibody against a common epitope detected recombinant *P. falciparum, P. vivax* and *P. yoelii* and native *P. falciparum* and *P. vivax* protein lactate dehydrogenase. Antibodies against species specific lactate dehydrogenase epitopes differentiated between *P. falciparum* and *P. vivax* lactate dehydrogenase. The study supports an anti-peptide antibody approach for the design of malaria diagnostic reagents.

